# When blood hits the brain: altered glymphatic and dural lymphatic function after surface bleeds

**DOI:** 10.1007/s00701-025-06760-9

**Published:** 2026-01-05

**Authors:** Per Kristian Eide, Markus Hovd, Lars Magnus Valnes, Are Pripp, Geir Ringstad

**Affiliations:** 1 Department of Neurosurgery, Oslo University Hospital—Rikshospitalet, Oslo, Norway; 2 Institute of Clinical Medicine, Faculty of Medicine, University of Oslo, Oslo, Norway; 3 K.G. Jebsen Centre for Brain Fluid Research, University of Oslo, Oslo, Norway; 4 Section for Pharmacology and Pharmaceutical Biosciences, Department of Pharmacy, University of Oslo, Oslo, Norway; 5 Department of Transplantation Medicine, Oslo University Hospital, Oslo, Norway; 6 Department of Mathematics, University, Oslo, Norway; 7 Oslo Centre of Biostatistics and Epidemiology, Research Support Services, Oslo University Hospital, Oslo, Norway; 8 Department of Radiology, Oslo University Hospital—Rikshospitalet, Oslo, Norway; 9 Department of Geriatrics and Internal Medicine, Sørlandet Hospital, Arendal, Norway

**Keywords:** Acute subdural hematoma, Subarachnoid hemorrhage, Glymphatic function, Dural lymphatic function

## Abstract

**Background:**

The impact of acute subdural hematoma (aSDH) on measures of glymphatic–meningeal lymphatic function has not previously been reported. We present a descriptive observational study including a small case series—one patient following unilateral aSDH, three following unilateral subarachnoid hemorrhage (SAH), and three control subjects—providing new insights into the differential effects of surface intracranial bleeds.

**Methods:**

The magnetic resonance imaging (MRI) contrast agent gadobutrol (0.5 mmol), administered intrathecally, was used as a cerebrospinal fluid (CSF) tracer. Multiphase contrast-enhanced MRI was performed to assess glymphatic tracer enrichment. CSF tracer clearance to blood, serving as a proxy for dural lymphatic function, was estimated using population pharmacokinetic modeling. All hemorrhagic cases involved unilateral bleeds, allowing within-subject comparison between affected and unaffected hemispheres.

**Results:**

The series included one patient with aSDH (2.8 months post-event), three patients with unilateral SAH (mean 5.8 months post-event), and three age-matched, near-healthy reference subjects. Compared with controls, glymphatic tracer enrichment 24 h post-injection was slightly increased on the affected hemisphere in the aSDH case, whereas SAH patients showed markedly reduced enrichment on the affected side. Tracer distribution in controls was symmetrical. CSF clearance to blood was notably reduced in the aSDH case compared with references, suggesting impaired dural lymphatic function.

**Conclusion:**

This small descriptive series suggests that aSDH and SAH may differentially affect glymphatic and dural lymphatic functions. While glymphatic enrichment appeared only modestly altered after aSDH, it was severely impaired following SAH. In contrast, CSF clearance to blood was markedly reduced in the aSDH case, potentially reflecting compromised dural lymphatic drainage. The limited number of cases prevent broad generalization, but these findings offer novel hypothesis-generating observations that may inform future studies on the effects of surface intracranial hemorrhages on brain clearance pathways.

## Introduction

An increasing body of evidence underscores the crucial role of the glymphatic and meningeal lymphatic systems in fluid and solute transport and clearance within the human brain (for review, see [11, 18]). While subarachnoid hemorrhage (SAH) is known to markedly impair glymphatic influx in humans [6], the effects of other types of intracranial hemorrhages on glymphatic and dural lymphatic function remain largely unexplored. In particular, the impact of acute or chronic subdural hematoma (SDH) has not yet been reported.

Experimental data addressing this topic are scarce. In one study, a pig with idiopathic acute subdural hematoma (aSDH) showed significantly impaired glymphatic transport [25]. Conversely, a mouse model of post-stroke brain edema demonstrated that early and enhanced cerebrospinal fluid (CSF) influx along glymphatic pathways exacerbated brain swelling [15]. Furthermore, unilateral craniectomy in mice—an intervention sometimes required in stroke patients—was associated with impaired glymphatic function, gliosis, and neurological deficits [17]. Collectively, these findings suggest that glymphatic function may be differentially affected by various types of brain injury and stroke.

In the present descriptive case series with controls, we report findings from one patient who experienced unilateral aSDH approximately three months before undergoing intrathecally enhanced glymphatic MRI (gMRI). We evaluated how CSF tracer enrichment differed between the affected and unaffected hemispheres and compared these results with three age-matched, near-healthy reference subjects. In addition, observations from this aSDH case were compared with data from three patients who had previously suffered unilateral SAH. CSF tracer clearance to blood, used as a proxy for meningeal lymphatic drainage capacity [3, 10], was also assessed in the aSDH patient and compared with reference data.

Preliminary findings from this study indicate that following aSDH, dural lymphatic function may be more substantially compromised than glymphatic function.

## Materials and methods

### Case series with controls

This study included patients who were hospitalized at the Department of Neurosurgery, Oslo University Hospital–Rikshospitalet (Oslo, Norway), and underwent glymphatic MRI (gMRI) based on clinical indications of suspected cerebrospinal fluid (CSF) disturbances. At the time of gMRI, none of the patients had external CSF drainage in place or were receiving treatment for hydrocephalus. All examinations followed an identical MRI protocol and were performed on the same scanner. From our gMRI database, this case series comprised one patient with aSDH, three patients with unilateral SAH, and three sex- and age-matched control subjects.

### Glymphatic magnetic resonance imaging (gMRI)

Glymphatic enrichment was visualized and semi-quantified using standardized 3D T1-weighted volume MRI scans performed before and 24 h after the intrathecal injection of gadobutrol (0.50 mmol, 0.50 mL of 1.0 mmol/mL; Gadovist, Bayer Pharma AG, Berlin, Germany) as a CSF tracer. Imaging was conducted using a 3 Tesla Philips Ingenia MRI scanner (Philips Medical Systems, Best, The Netherlands) equipped with a 32-channel head coil. Identical imaging parameters were applied across all scans: repetition time = "shortest" (typically 5.1 ms), echo time = "shortest" (typically 2.3 ms), flip angle = 8 degrees, field of view = 256 × 256 mm, and matrix = 256 × 256 pixels (reconstructed to 512 × 512). Each scan acquisition lasted 6 min and 29 s. Gadobutrol shortens the T1 relaxation time of water, enhancing T1 signal intensity in the grayscale images. This percentage change served as a semi-quantitative measure of tracer concentration.

Post-processing of the MRI data was performed using FreeSurfer software (version 6.0; http://surfer.nmr.mgh.harvard.edu/). FreeSurfer was employed for segmentation, parcellation, and alignment of longitudinal data. Segmentation and parcellation identified changes in T1 signal intensity caused by the CSF tracer [7]. These methods included removal of non-brain tissue using a hybrid watershed/surface deformation approach [23] and automated Talairach transformation. Subcortical white matter and deep gray matter volumetric structures were segmented using established methods [8]. The median T1 signal intensity for each segmented area was calculated at each time point and normalized against a reference region of interest (ROI) placed in orbital fat tissue, which does not enhance with contrast. This normalization corrected for baseline variations in image grayscale between scans. Tracer enrichment was quantified as the percentage change in normalized T1 signal intensity at 24 h post-injection. The subarachnoid space (SAS) was segmented using co-registered T2-weighted volume scans and parcellated based on the nearest and most frequent FreeSurfer labels.

### CSF clearance

To evaluate CSF clearance, plasma samples were quantified for gadolinium and recalculated into concentrations of gadobutrol, as previously described [10]. A previously developed population pharmacokinetic model was applied to determine individual pharmacokinetic parameters for intrathecal gadobutrol [10].

### Statistical analysis

Glymphatic enrichment at 24 h is presented as median percentage change in normalized T1 signal intensity with 25th and 75th percentile for the segmented areas within each major brain region. Due to the small sample size in each patient category, no formal statistical analysis was conducted.

## Results

### Study group

This study included seven individuals who underwent gMRI between September 2017 and September 2021. An overview of the cohort is provided in Table 1.

**Table 1:** Information about patients

	Patient groups		
	aSDH	Reference	SAH
***Demographic***			
N	1	3	3
Sex (F/M)	0/1	1/2	2/1
Age (years)	73	71.0 ± 3.6	47.7 ± 9.3
***Bleed***			
Months from bleed	2.8	-	5.8 ± 2.0
Cause of bleed			
MCA aneurysm		-	2
AChA		-	1
Unknown	1	-	
***Treatment***			
Craniotomy	1		3
EVD in acute phase	1		3

Categorical variables presented as numbers, and continuous variables as average ± standard deviation. *AChA* Anterior choroidal artery, *aSDH* Acute subdural hematoma, *EVD* External ventricular drainage. *MCA* Middle cerebral artery, *REF* Reference subjects, *SAH* Subarachnoid hemorrhage

#### Acute subdural hematoma (aSDH)

**Fig. 1:**
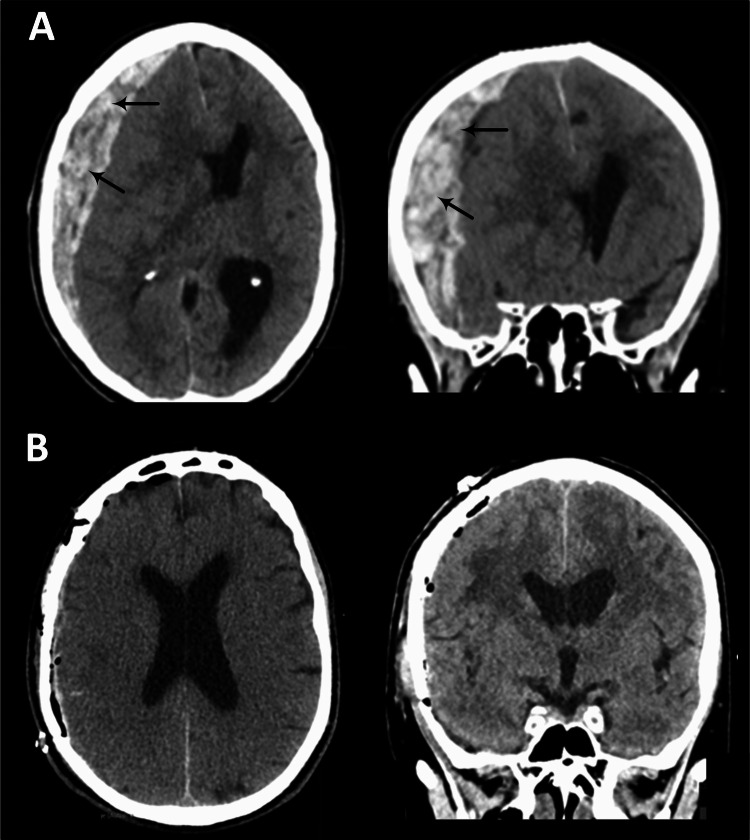
Acute subdural hematoma (aSDH) before and after craniotomy. A non-enhanced computed tomography (CT) scan in a 73-year-old man shows an aSDH (**A**) before craniotomy (black arrows), and (**B**) the day after craniotomy with evacuation of the bleed. The gMRI was performed 2.8 months after surgery. Axial (left) and coronal (right) CT scans are shown

A 73-year-old man presented with a spontaneous, non-traumatic acute subdural hematoma and a Glasgow Coma Scale (GCS) score of 7 on admission. No underlying vascular malformation was detected, and the cause of the spontaneous bleed remained unidentified. A computed tomography (CT) scan showing the hematoma is displayed in Fig. 1A. The patient underwent immediate craniotomy with dural opening and hematoma evacuation. A postoperative CT scan is shown in Fig. 1B. An external ventricular drain (EVD) was placed for cerebrospinal fluid (CSF) diversion. The patient was hospitalized for 13 days before being transferred to rehabilitation. gMRI was performed 2.8 months after the bleed. At that time, he had recovered well and was independently mobile, though he reported subjective cognitive difficulties that developed after the hemorrhage.

#### Reference subjects (REF)

The cohort included three control individuals matched to the aSDH patient by age and sex. These subjects underwent gMRI due to suspected CSF disturbances; however, comprehensive diagnostic evaluations revealed no evidence of neurological disease or CSF abnormalities. As intrathecal gadobutrol administration is off-label and requires a lumbar puncture, fully healthy volunteers were not included. Accordingly, these reference (REF) subjects are considered as close to healthy as possible.

#### Subarachnoid hemorrhage (SAH)

For comparison, three patients who had experienced unilateral SAH were included, with an average interval of 5.8 months between the hemorrhage and gMRI. The SAH was caused by a middle cerebral artery (MCA) aneurysm in two patients and by an anterior choroidal artery (AChA) aneurysm in one. All aneurysms were treated surgically by craniotomy and clip ligation. Each patient had an EVD during the acute phase but, at the time of gMRI, none had external CSF drainage or ongoing treatment for hydrocephalus. In all three cases, the initial hemorrhage was asymmetrically distributed and confined to one hemisphere (the affected side), facilitating comparison with the unilateral aSDH case.

### Tracer enrichment in CSF spaces

**Fig. 2:**
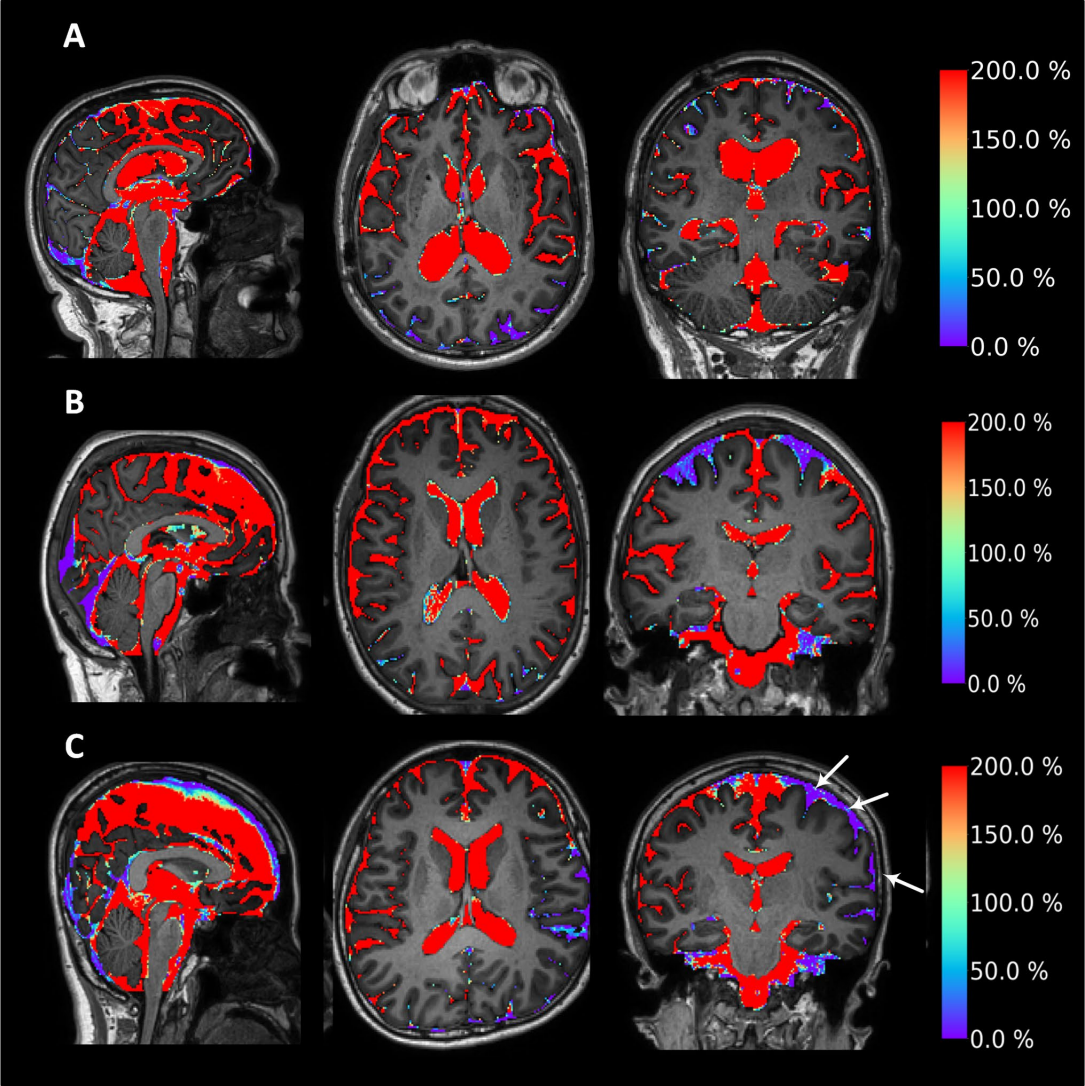
Impact of cerebral bleed on tracer enrichment in subarachnoid spaces and ventricles 24 h after intrathecal tracer injection. Tracer enrichment in the subarachnoid spaces was symmetrical between (**A**) the affected and unaffected hemispheres of the patient with acute subdural hematoma (aSDH) 2.8 months earlier and between (**B**) the left and right hemispheres of a reference (REF) subject. In contrast, **C** tracer enrichment was reduced on the affected side (white arrows) in a subject with asymmetrical subarachnoid hemorrhage (SAH) 6.3 months earlier (i.e., reduced tracer enrichment on affected side). All three cases exhibited strong ventricular tracer enrichment at 24 h. The degree of tracer enrichment 24 h after intrathecal injection is displayed in sagittal (left column), axial (middle column), and coronal (right column) MR images. The percentage increases in tracer enrichment compared to baseline are indicated on the color bar to the right

**Fig. 3:**
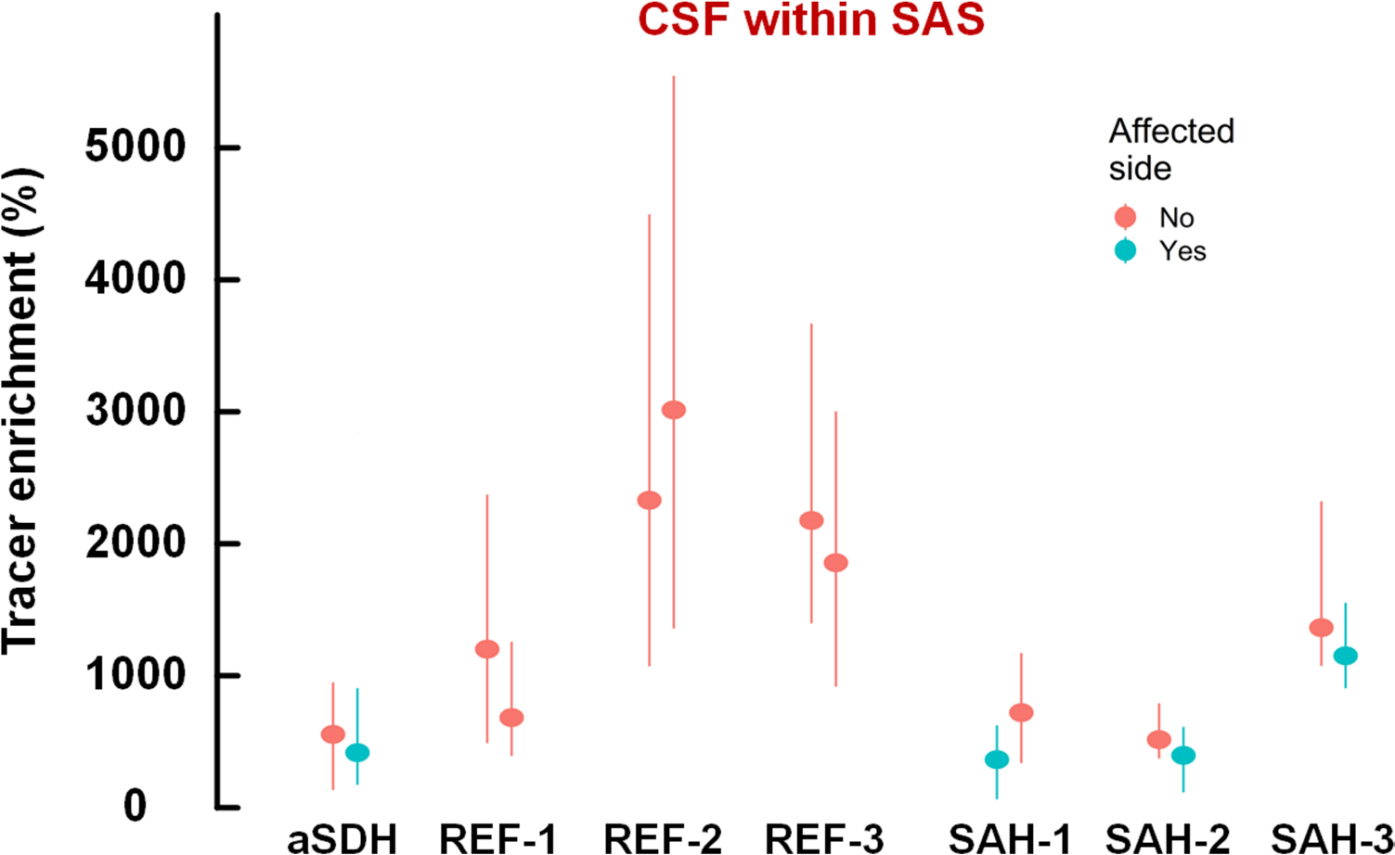
Comparisons of tracer enrichment in subarachnoid spaces over affected and unaffected hemispheres after intracranial bleeds. Tracer enrichment at 24 h within the CSF of the entire supratentorial subarachnoid space is shown for the individual with acute subdural hematoma (aSDH), the reference subjects (REF-1, REF-2, and REF-3), and the three subjects with asymmetrical subarachnoid hemorrhage (SAH-1, SAH-2, and SAH-3). In subjects with a previous intracranial bleed (aSDH or SAH), the degree of tracer enrichment somewhat lower over the affected hemisphere. Data are presented as median (dots) with 25th and 75.^th^ percentile (vertical lines)

Tracer enrichment in the subarachnoid and ventricular spaces 24 h after intrathecal tracer administration is shown in Fig. 2. When considering the entire subarachnoid space (SAS) bilaterally, tracer enrichment was reduced in subjects with previous aSDH or SAH compared to reference subjects. Within the patient groups, enrichment was slightly lower over the affected hemisphere in both aSDH and SAH cases (Fig. 3). All study participants exhibited tracer reflux, grade 3–4, into the cerebral ventricles, as illustrated in Fig. 2.

### Measures of glymphatic enrichment

**Fig. 4:**
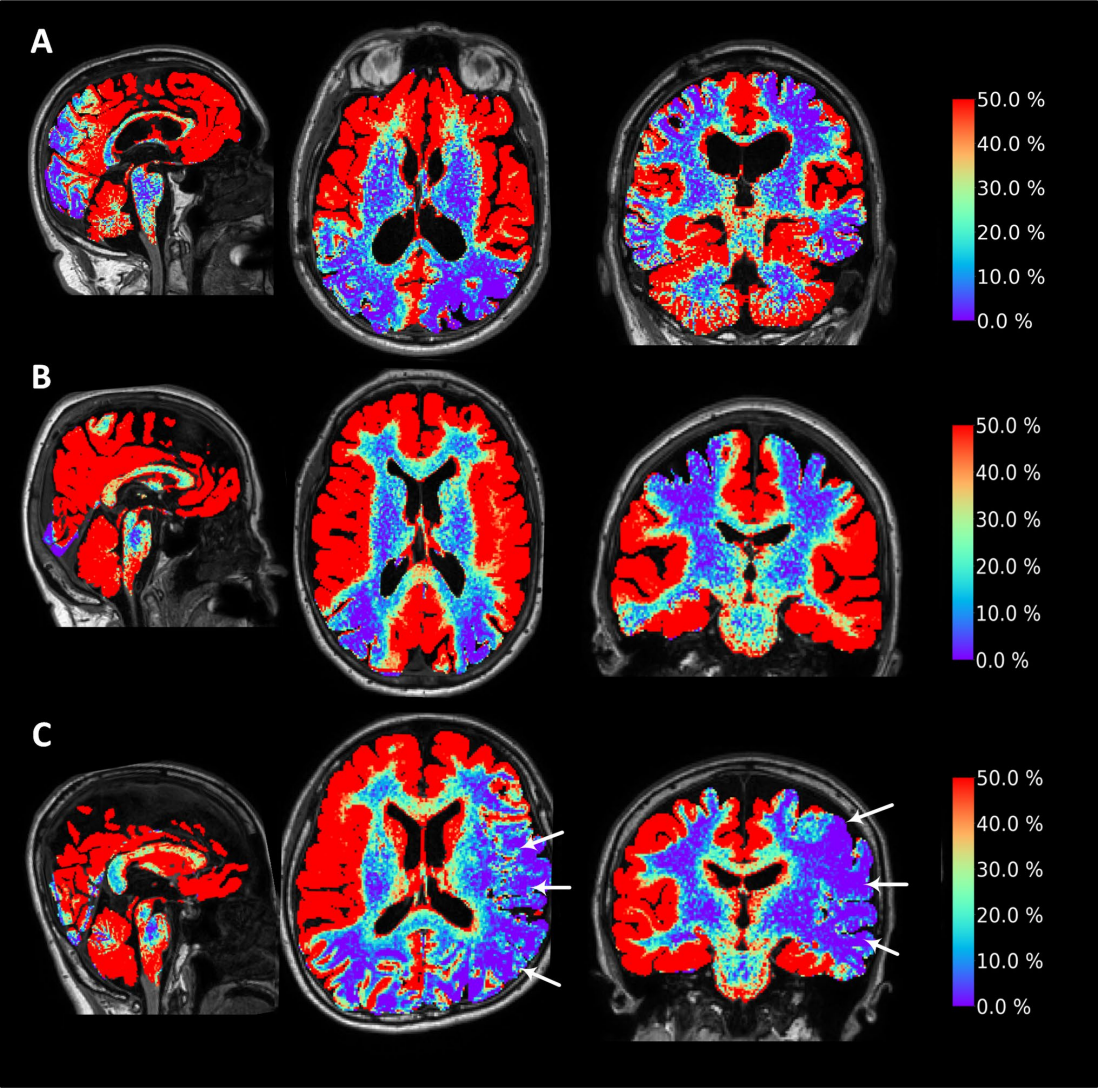
Impact of cerebral bleeds on glymphatic enrichment 24 h after intrathecal tracer injection. Glymphatic enrichment was symmetrical between (**A**) the affected and unaffected hemispheres of the subject with acute subdural hematoma (aSDH) 2.8 months earlier, and (**B**) between the left and right hemispheres in a reference (REF) subject. In contrast, **C** glymphatic enrichment was asymmetrically and markedly reduced on the affected side (white arrows) in a subject with asymmetrical subarachnoid hemorrhage (SAH) 6.3 months earlier. The degree of glymphatic enrichment 24 h after intrathecal tracer injection is illustrated in sagittal (left column), axial (middle column), and coronal (right column) MR images. Percentage increases in tracer enrichment compared to baseline are shown on the color bar to the right

**Fig. 5:**
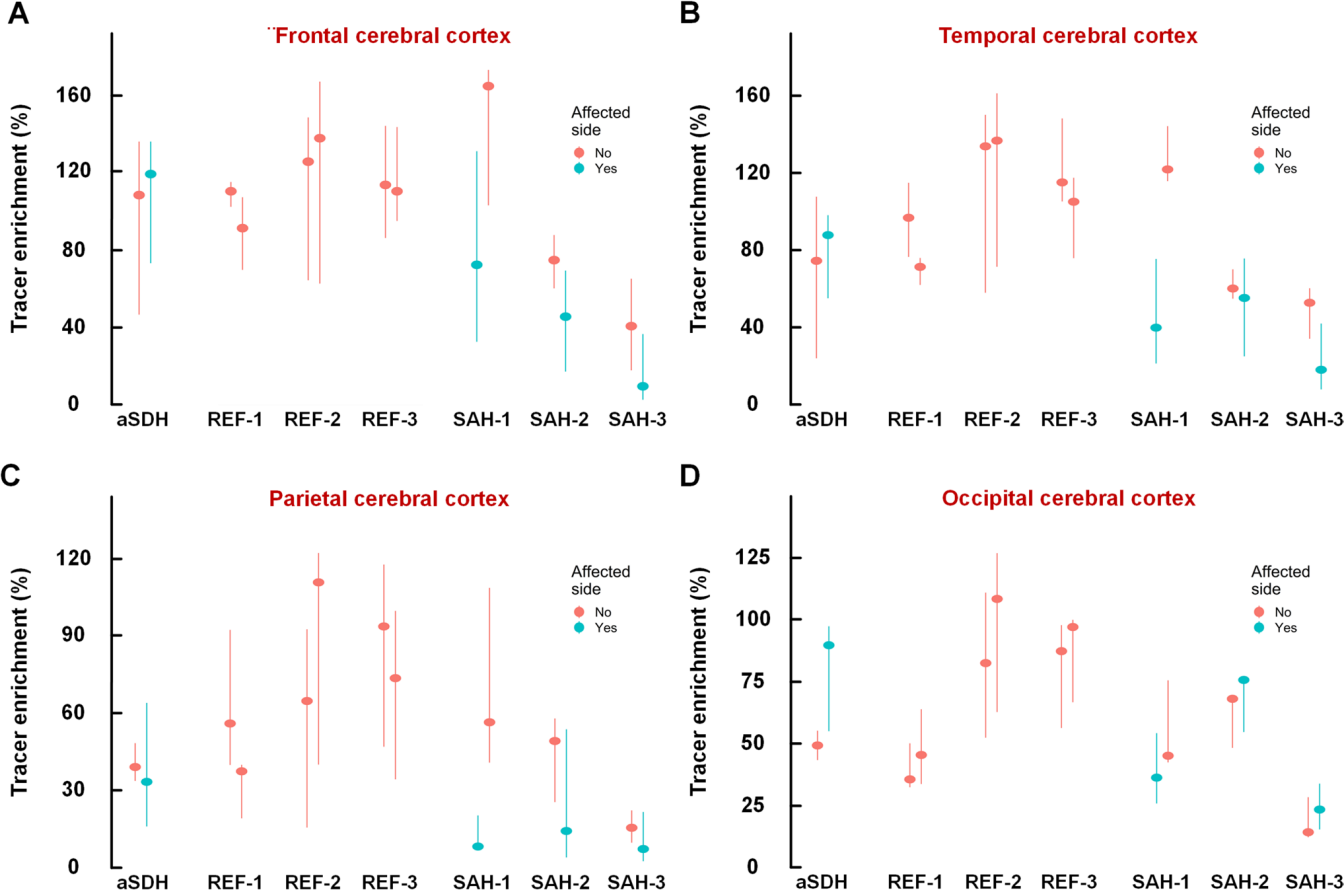
Comparisons of tracer enrichment in affected and unaffected cerebral cortex after intracranial bleeds. Tracer enrichment at 24 h in cerebral cortex is shown for the individual with acute subdural hematoma (aSDH), reference subjects (REF-1, REF-2, and REF-3), and the three subjects with asymmetrical subarachnoid hemorrhage (SAH-1, SAH-2, and SAH-3). Results for the cerebral lobes (**A**) frontal cortex, **B** temporal cortex, **C** parietal cortex and (**D**) occipital cortex are displayed. In the subject with acute subdural hematoma (aSDH) 2.8 months earlier, tracer enrichment tended to be somewhat higher in cerebral cortex at affected side (except for in parietal cortex). In contrast, the individuals with asymmetrical SAH average 5.8 months before, tracer enrichment was generally lower in cerebral cortex of affect side (except for occipital cortex in two subjects). Data are presented as median (dots) with 25th and 75.^th^ percentile (vertical lines)

Glymphatic enrichment, expressed as the percentage increase in tracer concentration at 24 h, is visualized for a representative case from each group in Fig. 4. In the aSDH patient, glymphatic enrichment within the cerebral cortex was generally slightly increased in the affected hemisphere, whereas REF subjects showed no systematic interhemispheric differences (Fig. 5A–D). In contrast, the three SAH patients exhibited markedly reduced cortical glymphatic enrichment on the affected side compared with the contralateral hemisphere (Fig. 5A–D).

### Measures of dural lymphatic function

**Fig. 6:**
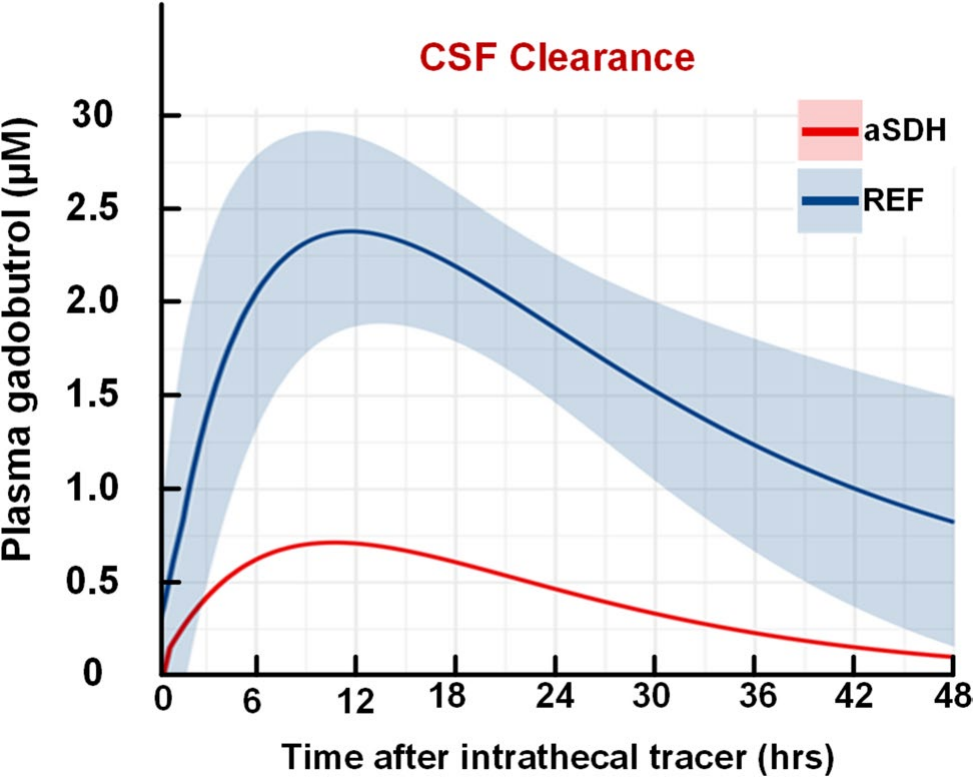
Impaired CSF clearance after acute subdural hematoma (aSDH). Predicted plasma concentrations of the CSF tracer (gadobutrol) over time for the aSDH patient (red line), compared to the age-matched reference (REF) subjects. The REF group is represented by the average and 95% confidence intervals (CI)

Measures of CSF clearance, serving as a proxy for meningeal lymphatic function, indicated markedly impaired clearance from CSF to blood in the aSDH patient compared with REF subjects (Fig. 6). CSF clearance data were not available for the SAH cases.

For the aSDH patient, the time to 50% clearance of tracer from CSF to blood (T½) was 7.4 h, compared with 5.6 ± 3.4 h in the REF cohort. The maximum plasma tracer concentration (C_max_) was 0.7 µM at 10.6 h post-administration, compared with 2.4 µM at 11.1 h in the REF cohort. The apparent lag time (T_lag_; i.e., time before clearance process starts) was 0.45 h in the aSDH patient versus 0.74 h among reference subjects.

## Discussion

The present preliminary findings suggest differing patterns of glymphatic function between the subject with prior aSDH and those with previous unilateral SAH. Even though broad generalization cannot be made, the observations from this single aSDH case indicate that aSDH may have a relatively limited impact on glymphatic function but a more pronounced effect on dural lymphatic clearance when compared with reference subjects.

To our knowledge, this is the first study to investigate the impact of aSDH on glymphatic–meningeal lymphatic function in humans. The only prior work in this area involved a pig with idiopathic aSDH, where the acute hematoma caused immediate and severe impairment of glymphatic tracer transport [25]. The extent to which normal anatomy and function are restored after craniotomy and hematoma evacuation remains uncertain. In the present case, gMRI was performed 2.8 months after the hemorrhage. It is likely that glymphatic function was impaired during the acute phase; however, because aSDH is located outside the subarachnoid space, it is reasonable to hypothesize that glymphatic influx partially normalized after evacuation. In this aSDH case, no underlying cause was identified, which is uncommon. However, the hematoma was large (Fig. 1), enabling the exploration of the impact of aSDH.

Acute SDH originates at the interface between the arachnoid barrier cell (ABC) layer and the dural border cell (DBC) layer [14]. There is no natural subdural space [9]; rather, this potential space forms as a consequence of pathological conditions such as SDH. Rodent studies have demonstrated lymphatic vessels within the dura that contribute to CSF drainage [1, 13], particularly in the parasagittal region, but also more laterally at the convexities [27]. A hemorrhage that directly involves the dura could therefore disrupt dural lymphatic clearance. The slightly elevated CSF tracer levels in the brain 24 h after tracer injection in the aSDH patient (relative to references) may reflect reduced CSF clearance, as the system is well into the clearance phase by this time point [28]. Previous studies have shown that CSF clearance is a major determinant of overall brain clearance [22], and that glymphatic outflow depends directly on lymphatic CSF clearance capacity [21].

**Fig. 7:**
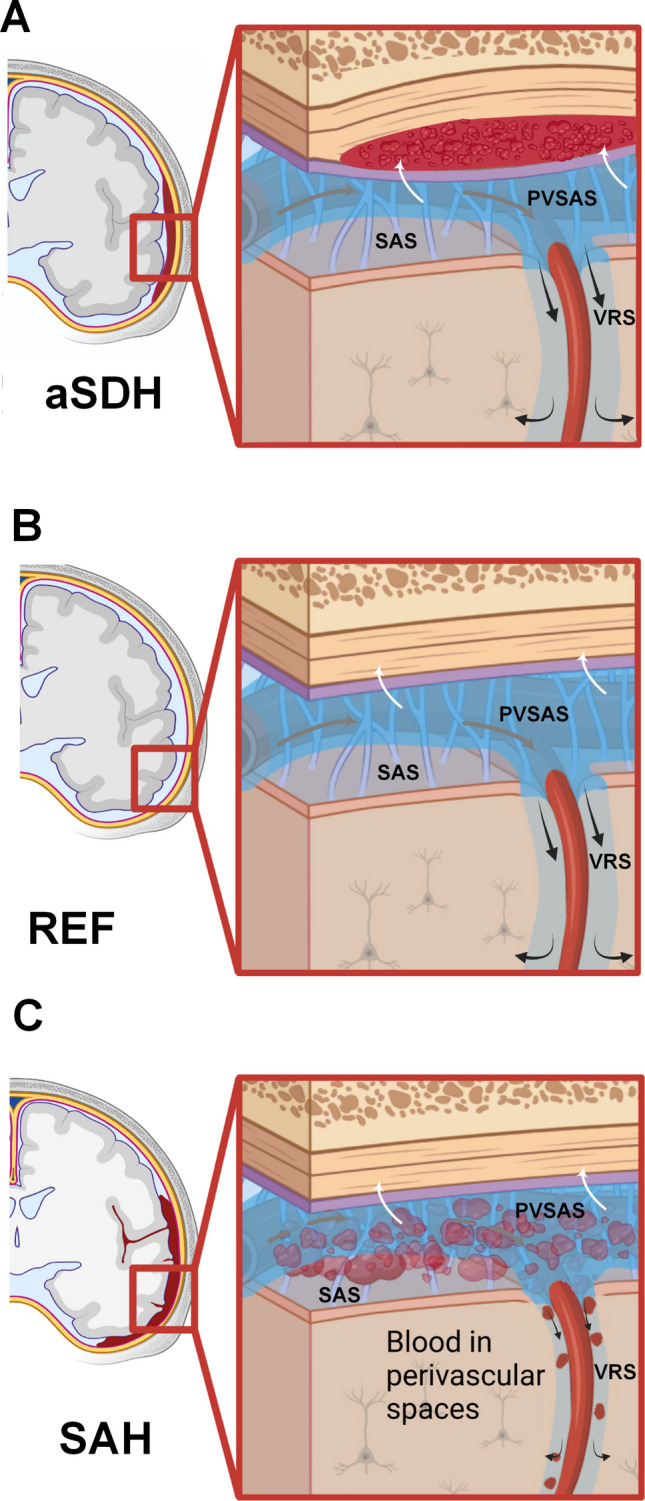
Illustration of possible different effects of acute subdural hematoma (aSDH) and subarachnoid hemorrhage (SAH) on glymphatic and dural lymphatic functions. A diagram illustrates our hypothesis on how solute (tracer) transport is affected by different types of intracranial bleeds. **A** Following aSDH, the perivascular subarachnoid spaces (PVSAS) and cortical Virchow-Robin perivascular spaces (VRS) may not be obstructed, thereby not preventing solute transport within PVSAS (gray arrows). However, the movement of solutes across the arachnoid barrier layer toward the dura (white arrows) may be impaired, possibly due to inflammation in the meninges created by the bleed, which may restrict transdural tracer passage, potentially affecting dural lymphatic function. In (**A**), the subdural blood clot is shown but was removed in our case 2.8 months prior to gMR. However, the passage across dura may also be affected after removal of bleed.** B** Under normal conditions, as in the present reference (REF) subjects, solute (tracer) transport in the CSF (gray arrows) is facilitated along the perivascular subarachnoid spaces (PVSAS) toward the parenchymal Virchow-Robin perivascular spaces (VRS; black arrows). Substances in the subarachnoid space (SAS), outside the PVSAS, may pass towards the dura mater, where lymphatic vessels reside (white arrows). **C** Subarachnoid hemorrhage (SAH) was shown to impair the function of both the PVSAS and cortical VRS, limiting glymphatic tracer enrichment in the brain. Experimental data suggest that meningeal clearance function may also be compromised by SAH. Illustration: Cesar Luis Vera Quesada, Oslo University Hospital

The markedly impaired glymphatic enrichment observed in SAH patients is most likely secondary to disturbed CSF tracer propagation in the subarachnoid space, as enrichment at the brain surface is a prerequisite for tracer penetration into brain tissue [22]. SAH may obstruct the perivascular subarachnoid space (PVSAS) (Fig. 7), previously shown to facilitate CSF inflow toward the brain [4]. Thus, the reduced glymphatic enrichment on the affected side in SAH patients aligns with earlier reports demonstrating that SAH severely disrupts glymphatic transport [6].

Both the aSDH and SAH subjects underwent craniotomy with aneurysm clip surgery in the three SAH patients in accordance with institutional practice [5]. The potential impact of craniotomy itself on glymphatic or dural lymphatic function remains less studied, except for the one experimental study in mice that reported impaired glymphatic function following hemicraniectomy [17]. However, within this small series, the craniotomy can hardly explain the observed differences between the aSDH and SAH cases. Future studies could compare intracranial surface bleeds treated endovascularly, surgically, or conservatively.

In this study, glymphatic enrichment was assessed 24 h after intrathecal tracer administration, reflecting a combination of tracer influx and clearance. Imaging at later time points—such as 48–72 h post-injection—could provide more specific data on clearance dynamics. At 24 h, the enrichment phase predominates; therefore, the term glymphatic enrichment was used rather than glymphatic clearance.

Two main hypotheses have been proposed for CSF efflux pathways: (1) through bridging veins that traverse the dura along arachnoid cuff exits [26] and (2) via arachnoid granulations projecting into the dura mater [24]. aSDH is of particular interest because it may disrupt both structures, potentially impairing CSF efflux. Tracer studies have demonstrated direct movement of CSF tracer into the parasagittal dura [19], and skull bone marrow [20]. Subdural hematoma could therefore serve as a model for studying how pathological processes alter these normal efflux routes between the cortex and meninges. The markedly reduced CSF elimination rate in the aSDH patient is consistent with this hypothesis. Nevertheless, it remains unclear why a unilateral aSDH confined to one hemisphere (Fig. 1A) would cause a global impairment of CSF efflux, as compensatory drainage pathways should theoretically mitigate such effects. Further studies are needed to clarify this mechanism.

The principal long-term symptom reported by the aSDH patient was cognitive dysfunction, despite resolution of neurological deficits and normalization of structural MRI findings. While this may represent a delayed complication of the hemorrhage, an alternative explanation could involve impaired clearance of metabolic waste. Such dysfunction has been suggested as a common mechanism underlying abnormal protein aggregation in dementias [16] and measures of glymphatic-meningeal function associate with blood biomarkers of neurodegeneration and dementia [3]. Intracranial hemorrhages are frequently associated with persistent fatigue and cognitive impairment [29], and both ischemic and hemorrhagic stroke increase the risk of dementia [2]. Impaired CSF clearance could therefore contribute to cognitive sequelae, although this remains speculative and requires further investigation.

## Limitations

Several limitations should be acknowledged. First, the number of subjects was small, and findings must therefore be regarded as preliminary. Second, the temporal resolution of gMRI was limited, which may constrain interpretation of clearance kinetics. Histological analysis of dural tissue would have provided complementary information but was not available. Third, the time from ictus to imaging varied with longer recovery time in the SAH cases; however, based on previous results [6], even more pronounced effects of SAH would be expected shorter after ictus. Despite these limitations, the study’s strengths include standardized imaging protocols and CSF clearance measurements, as well as comparison with age-matched reference subjects—important given the known age-dependence of glymphatic function [12].

Moreover, as all hemorrhagic cases had undergone craniotomy, it cannot be determined to what extent the observed alterations reflect the surface bleed itself or surgical intervention.

## Conclusions

This small descriptive case series provides preliminary observations that may guide future research. The findings suggest that unilateral aSDH has only a modest long-term effect on glymphatic enrichment, in contrast to the pronounced impairment observed months following SAH. However, aSDH (and possibly craniotomy) appears to markedly reduce CSF clearance, likely due to disrupted dural lymphatic function. Why this reduction is not compensated for through alternative CSF efflux routes remains uncertain and warrants further investigation in larger, systematic studies.

## Data Availability

The authors are willing to make their data, analytic methods, and study materials available to other researchers. Corresponding author is contact person.

## Abbreviations

aSDH: Acute subdural hematoma
CSF: Cerebrospinal fluid
CT: Computed tomography
EVD: External ventricular drainage
gMRI: Glymphatic MRI
MRI: Magnetic resonance imaging
SAH: Subarachnoid hemorrhage
REF: References
